# Changes of interictal epileptiform discharges during medication withdrawal and seizures: A scalp EEG marker of epileptogenicity

**DOI:** 10.1016/j.cnp.2022.09.004

**Published:** 2022-10-07

**Authors:** Pia De Stefano, Eric Ménétré, Serge Vulliémoz, Pieter Van Mierlo, Margitta Seeck

**Affiliations:** a EEG and Epilepsy Unit, University Hospitals of Geneva, Geneva, Switzerland; b Neuro-Intensive Care Unit, Department of Intensive Care, University Hospitals of Geneva, Geneva, Switzerland; c Medical Image and Signal Processing Group, Department of Electronics and Information Systems, Ghent University, Ghent, Belgium

**Keywords:** EEG, Spike, Interictal discharges, Seizure, Seizure risk, Withdrawal, Anti-epileptic drugs, Anti-seizure medication, Circadian profile

## Abstract

**Objective:**

To determine the influence of antiseizure medication (ASM) withdrawal on interictal epileptogenic discharges (IEDs) in scalp-EEG and seizure propensity.

**Methods:**

We included 35 adult unifocal epilepsy patients admitted for presurgical evaluation in the EEG and Epilepsy Unit of Geneva between 2016 and 2020, monitored for at least 5 days. ASM was individually tapered down, and automated IED detection was performed using Epilog PreOp (Epilog NV, Belgium, Ghent). We compared spike rate per hour (SR) at day 1 when patients were on full medication (baseline) with SR at the day with the lowest dose of medication. To determine possible peri-ictal changes of SR, we compared SR 8 h before and after a seizure with the SR at the same time of the baseline day.

**Results:**

Our results showed a significant increase in spiking activity in the day of lowest drug load if compared to spike rate at day on full medication (p < 0.001). The total amount of spikes during 24 h correlated significantly with seizure occurrence (p < 0.0001). We also revealed significant increase in peri-ictal SR, in particular 2–4 h preceding a seizure (p = 0.05) extending up to 3 h after the seizure (p = 0.03) with a short decrease just before seizure occurrence.

**Conclusions:**

Our results suggest that SR increases with medication withdrawal and particularly before and after seizures. There is a complex pattern of increase and decrease around seizure onset which explains divergent results in previous studies.

**Significance:**

Precise spike counting at similar circadian periods for a patient could help to determine the risk of seizure occurrence in a personalized fashion.

## Highlights

• IEDs increased during ASM withdrawal and even more before and after seizures.
• The higher the increase of IEDs during 24 h, the higher the likelihood of seizures.
• EEG controls should be carried out always at the same time to allow comparison.

## 1 Introduction

Antiseizure medication (ASM) withdrawal is an effective and reliable method to increase the number of habitual seizures and activate the epileptic focus for diagnostic purposes during the presurgical assessment of patients with focal epilepsy (Andersen et al., 2010). Interictal epileptogenic discharges (IEDs) are considered as a surrogate measure for seizure propensity as shown in studies with chronically implanted electrodes over years (Leguia et al., 2021). These results suggest that IED counts contain valuable information to estimate seizure risk and are not only a by-product of the epilepsy itself with no other information than that the patient suffers from epilepsy.

ASM withdrawal may serve as a model for decreased ASM intake or drug inefficiency as it is often observed in clinical practice, although the exact relationship between IED changes and seizure occurrence is unknown. Patients undergoing continuous video-scalp EEG monitoring in the context of presurgical evaluation provide a unique chance to study this relationship.

Most of the previous studies, investigating the relationship between IEDs and drug withdrawal looked exclusively at diagnostic intracranial EEG, often with changing montages throughout monitoring due to technical limitations. They reported a lack of increase or even decrease of spikes in scalp EEG when medication was tapered down, and a spike rate (SR) increase was considered to be the result of the occurrence of seizures (Goncharova et al., 2016, Gotman and Koffler, 1989, Gotman and Marciani, 1985, Spencer et al., 2008).

Moreover, intracranial studies overestimate the “epileptogenic charge”, i.e., they usually show much more frequent IEDs than in scalp EEG, including from areas remote to the focus, whose relevance for the outcome of the surgery is not well understood. In contrast, scalp EEG reflects the “raw” and primary focus or foci in a given patient, which makes scalp IEDs a very interesting marker of epileptogenicity.

Older scalp EEG studies suggested an inverse relationship between antiepileptic medication level and the number of IEDs (Ludwig and Ajmone Marsan, 1975, Milligan et al., 1983, Wilkus and Green, 1974), i.e. the lower the medication, the higher the frequency of IEDs. However, these results were obtained with EEGs of limited electrode counts (8–21 electrodes), potentially underestimating IED occurrence. In a more recent study, ASM withdrawal was carried out very abruptly or progressively leading to different IED changing patterns (Goncharova et al., 2016).

In the present study, we set out to explore the degree of changes of IEDs and their timing in relation to progressive ASM withdrawal in order to determine the value of scalp IEDs as possible marker for decreased or insufficient medication.

## 2 Methods

### 2.1 Patients

We identified all patients admitted for presurgical evaluation in the EEG and Epilepsy Unit of Geneva’s University Hospitals between 1.1 2016 and 31.12.2020. We included patients matching the following inclusion criteria: a) unifocal epilepsy patients, b) presence of interictal spikes in the EEG, c) patients > 16 years, d) if the entire long-term-EEG was stored and available for analysis. Exclusion criteria were: a) experiencing seizures already during the first day of EEG monitoring, b) status epilepticus during monitoring, c) a monitoring duration < 5 days.

We defined unifocal epilepsy if seizures arose from one focus and interictal epileptiform discharges in > 80 % from the same site.

In our centre, we use a standardized (progressive) protocol of ASM withdrawal with unchanged ASM the first day, allowing comparing IED rates with a baseline condition. ASM was tapered down individually based on their history of seizure frequency, the number and type of drugs, in order to minimize the risk of generalized seizures and status epilepticus (e.g. slower withdrawal with carbamazepine than with lamotrigine, or slower withdrawal of a specific drug if known to be crucial for seizure control in a determined patient). Our protocol stipulates progressive withdrawal, which starts at day 2 of the monitoring session. Each patient underwent monitoring video-EEG (Micromed Inc, Italy) with 38 scalp electrodes applied according to the 10–20 system. Periods without EEG recording did not exceed 1–2 h in total per patient (e.g., prolonged periods of personal hygiene).

### 2.2 Analyses

We considered the frequency of IEDs (“spike rate”, SR; IEDs/ hour) at day 1 of monitoring as baseline (“High” condition), i.e. the patient was on full medication. We compared “High” with SR on the days with the lowest dose of medication (“Low” condition). If patients had lowest or no medication over several days, we selected the first day of this period in order to homogenize comparisons across all patients. If a patient experienced a seizure, we analysed the entire day without the hour involving seizures to be sure that we include only interictal spikes.

To determine if the epileptic activity was modulated during the time around a seizure, we averaged IED activity in the 8 h before and after each seizure of each patient. Seizures occurring at an interval a less than 8 h interval were discarded. We compared SR with the same time period during baseline (e.g. if the seizure occurred at 11 pm the third day of monitoring, we compared the SR at day 1, 8 h before and after 11 pm).

The study has been approved by the ethical committee of the University Hospitals of Geneva.

### 2.3 IED detection and processing

Automated IED detection was performed using Epilog PreOp (Epilog NV, Belgium, Ghent), a framework for automatic detection of epileptic spikes. We identified the electrode where the spike maximum amplitude occurred. The average number of spikes was calculated per hour per cluster. Since the algorithms counts also spiky pattern, like wicket spikes, which are physiological, two EEG certified physicians (MS, PDS) verified the presence of epileptogenic foci, identified by the automatic detection algorithm. In the present study IEDs were not used for focus localization, but just as a marker of epileptogenic activity.

### 2.4 Statistics

To assess statistically the effect of drug withdrawal on interictal spike activity (coded as number of spikes per hour), we performed a generalized linear mixed model fitting a Gaussian distribution linked to a log function. The model included the time of measure as fixed factor and the patients’ ID as random intercept (accounting for the inter-individual variability and for the repeated measures effect). To overcome convergence issues, the optimizer was replaced by optimx (Nash and Varadhan, 2011) using the bobyqa method. Hence, differences between the averaged peri-ictal and baseline time window were analysed using a clustermass method. All the statistics and data wrangling were performed with the R software (R Core Team, 2020), using mainly the dplyr (Wickham et al., 2019), tidyr (Wickham and Henry, 2019) ggplot2 (Wickham, 2016), car (Fox and Weisbery, 2019), NPL (Ménétré, 2021), glmmTMB (Brooks et al., 2017), lme4 (Bates et al., 2015), permuco (Frossard and Renaud, 2021) and lmerTest (Kuznetsova et al., 2017) packages.

## 3 Results

35 patients fulfilled our inclusion and exclusion criteria (Table 1).

**Table 1:** Clinical data of patients. In column: M: male, F: female; %ASM after wtd = % of the lowest dose compared to baseline after withdrawal (0: no more medication); Day monitoring = day of monitoring with the lowest dose of medication; MRI = magnetic resonance imaging. HS = Hippocampal sclerosis, Post-CNS surgery = Post-central nervous system surgery; Aetiology: Non-Struct (Non-Structural); Localization = temporal or extratemporal focus; R = right hemispheric focus, L = left hemispheric focus; N. seizure = Number of Seizure per patient.

Patient	Age	Sex	% ASM after wtd	Day Monitoring	MRI	Etiology	Localization	Side	N. seizure
**1**	18	F	0	10	Normal	Non-Struct	Temporal	R	2
**2**	53	F	0	11	Normal	Non-Struct	Temporal	R	1
**3**	27	M	11.11	11	Normal	Non-Struct	Temporal	L	3
**4**	30	F	16.66	8	HS	Structural	Temporal	L	6
**5**	38	M	0	10	Normal	Non-Struct	Extratemporal	L	0
**6**	40	F	0	12	Normal	Non-Struct	Temporal	R	2
**7**	20	F	17	11	Normal	Non-Struct	Temporal	R	3
**8**	27	M	25	9	Dysplasia	Structural	Extratemporal	R	1
**9**	50	M	0	9	Meningioma	Structural	Temporal	R	1
**10**	39	M	0	11	Post-CNS surgery	Structural	Extratemporal	L	1
**11**	38	F	0	12	Normal	Non-Struct	Extratemporal	L	0
**12**	39	F	14.28	10	Normal	Non-Struct	Temporal	L	7
**13**	62	F	0	8	Dysplasia	Structural	Temporal	L	4
**14**	24	F	0	8	Normal	Non-Struct	Extratemporal	R	2
**15**	40	F	0	12	Normal	Non-Struct	Extratemporal	L	2
**16**	38	M	0	15	HS	Structural	Temporal	R	3
**17**	41	F	31.23	9	Normal	Non-Struct	Temporal	R	4
**18**	18	F	30	8	Dysplasia	Structural	Extratemporal	R	1
**19**	30	M	12.5	8	Normal	Non-Struct	Extratemporal	R	4
**20**	53	F	0	10	Radionecrosis	Structural	Temporal	R	2
**21**	42	F	0	8	Normal	Non-Struct	Temporal	R	3
**22**	48	M	25	5	HS	Structural	Temporal	L	0
**23**	34	M	37.5	6	Normal	Non-Struct	Extratemporal	R	2
**24**	57	F	27.7	5	Normal	Non-Struct	Temporal	R	2
**25**	48	F	12.5	6	HS	Structural	Temporal	R	1
**26**	47	F	0	8	Dysplasia	Structural	Extratemporal	R	3
**27**	49	M	0	12	Dysplasia	Structural	Extratemporal	R	2
**28**	30	F	33	8	HS	Structural	Temporal	L	2
**29**	23	M	21	10	Normal	Non-Struct	Temporal	L	4
**30**	53	M	0	10	Normal	Non-Struct	Temporal	R	3
**31**	16	F	0	7	Normal	Non-Struct	Temporal	L	3
**32**	48	F	22	7	Normal	Non-Struct	Extratemporal	L	2
**33**	57	F	47	8	Post-CNS surgery	Structural	Temporal	L	2
**34**	38	M	62	5	Brain Hemorrhage	Structural	Extratemporal	R	1
**35**	57	F	11	12	HS	Structural	Temporal	L	1

Duration of monitoring ranged from 5 to 15 days (median = 9 days IQR = 3). The median age was 39 (IQR = 18.5), 22 were females. Sixteen patients suffered from structural and 19 from non-structural epilepsy. 21 patients presented with temporal and 14 extra-temporal lobe epilepsy. Seizures were recorded in 32 out of 35 patients during the entire monitoring. A total of 80 seizures were recorded. The number of seizures for each patient ranged from 1 to 7 (median = 2, IQR = 2) (Table 2).

**Table 2:** Cumulative number of seizure occurrences during the monitoring period among patients.

Cumulative number of seizures	Number of patients
1 seizure	32
2 seizures	24
3 seizures	13
4 seizures	6
5 seizures	2
6 seizures	2
7 seizures	1

**Fig. 1:**
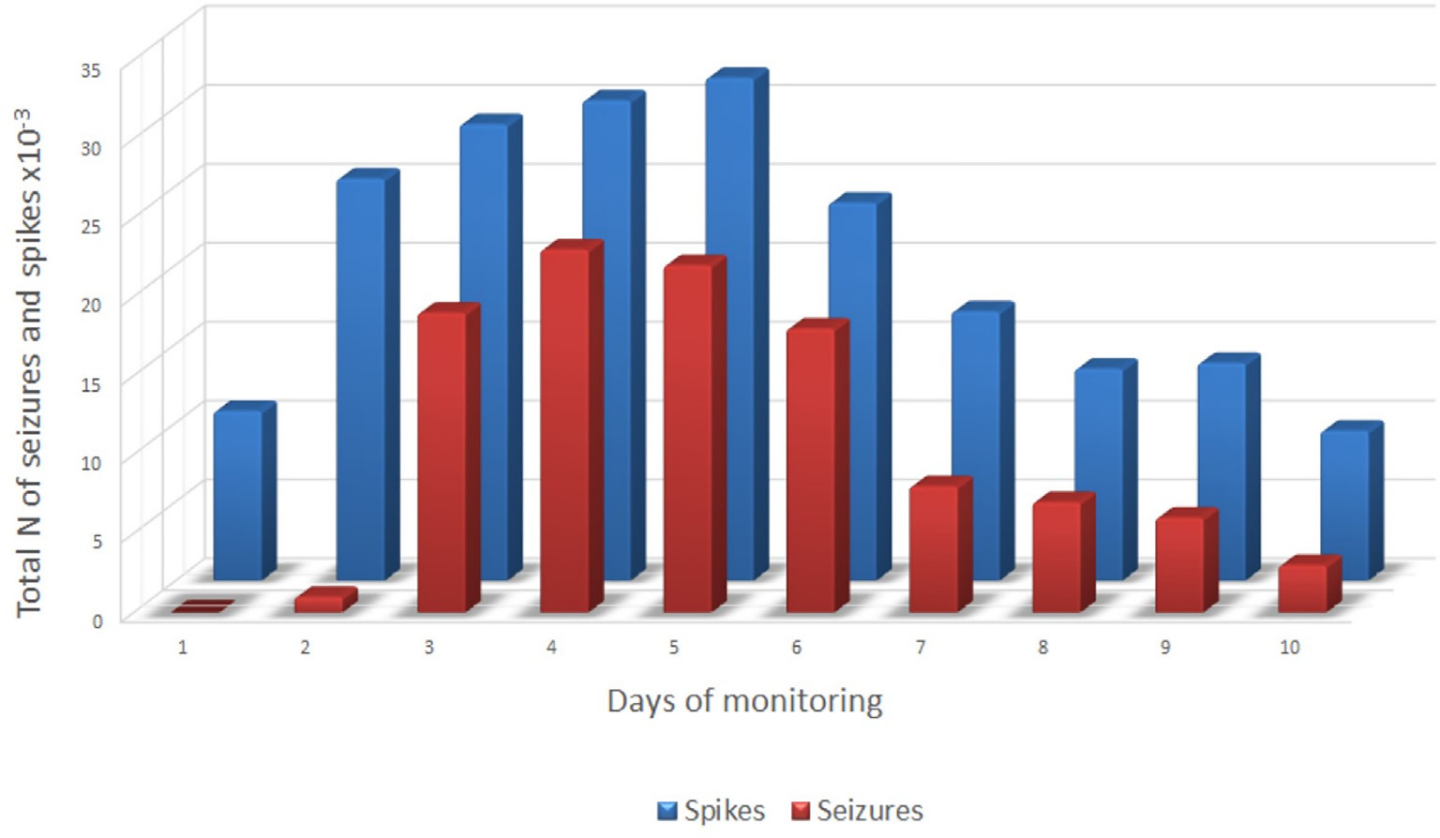
Relationship between interictal spiking and seizure occurrence. Total number of spikes activity × 10^−3^ across all patients (in blue) and total number of seizures across all patients (in red) per day of monitoring (not all patients reached 10 days of monitoring, see Table 1). Pearson correlation r 0.909 (p = 0.005). (For interpretation of the references to colour in this figure legend, the reader is referred to the web version of this article.)

The relationship between interictal spiking and the total number of seizure occurrence per day of the entire group is shown in Fig. 1.

The mean number of ASM taken at baseline was 2.54 (SD = 0.95) and at maximum withdrawal day was 0.77 (SD = 0.94), (*p* < 0.001).

**Fig. 2:**
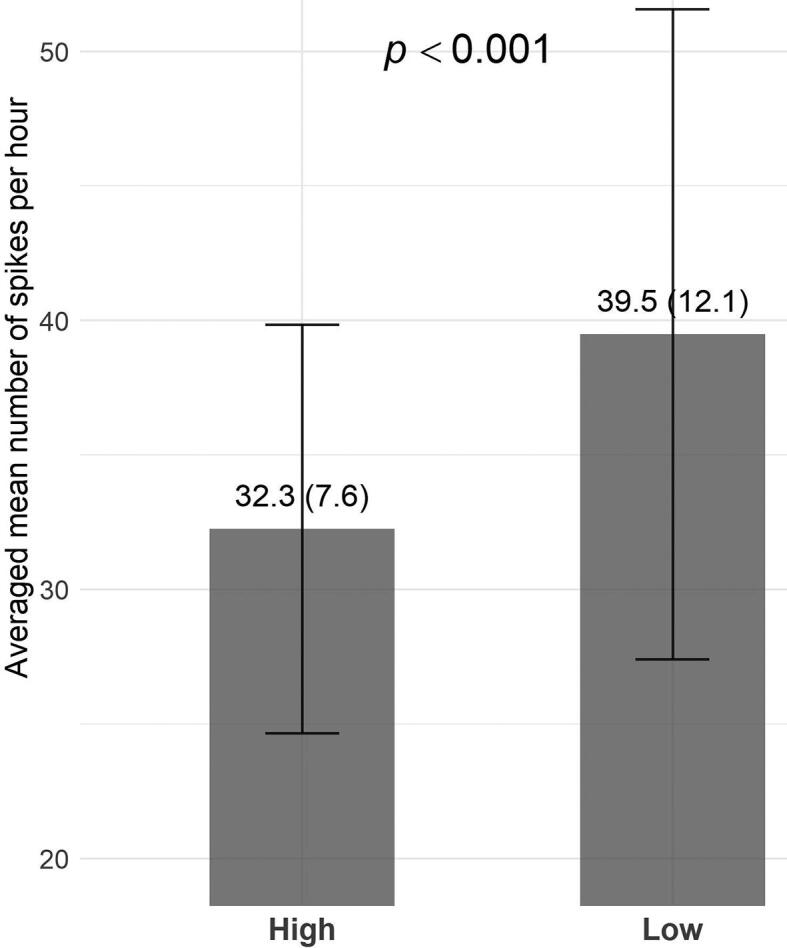
Comparison of spike charge during the 24 h of lowest medication compared to baseline. Mean number of spikes per hour averaged during baseline (day 1 – “High” condition-) when patients were on full dose medication and the day of the maximal withdrawal (“Low” condition). Error bars represent standard error of mean.

**Fig. 3:**
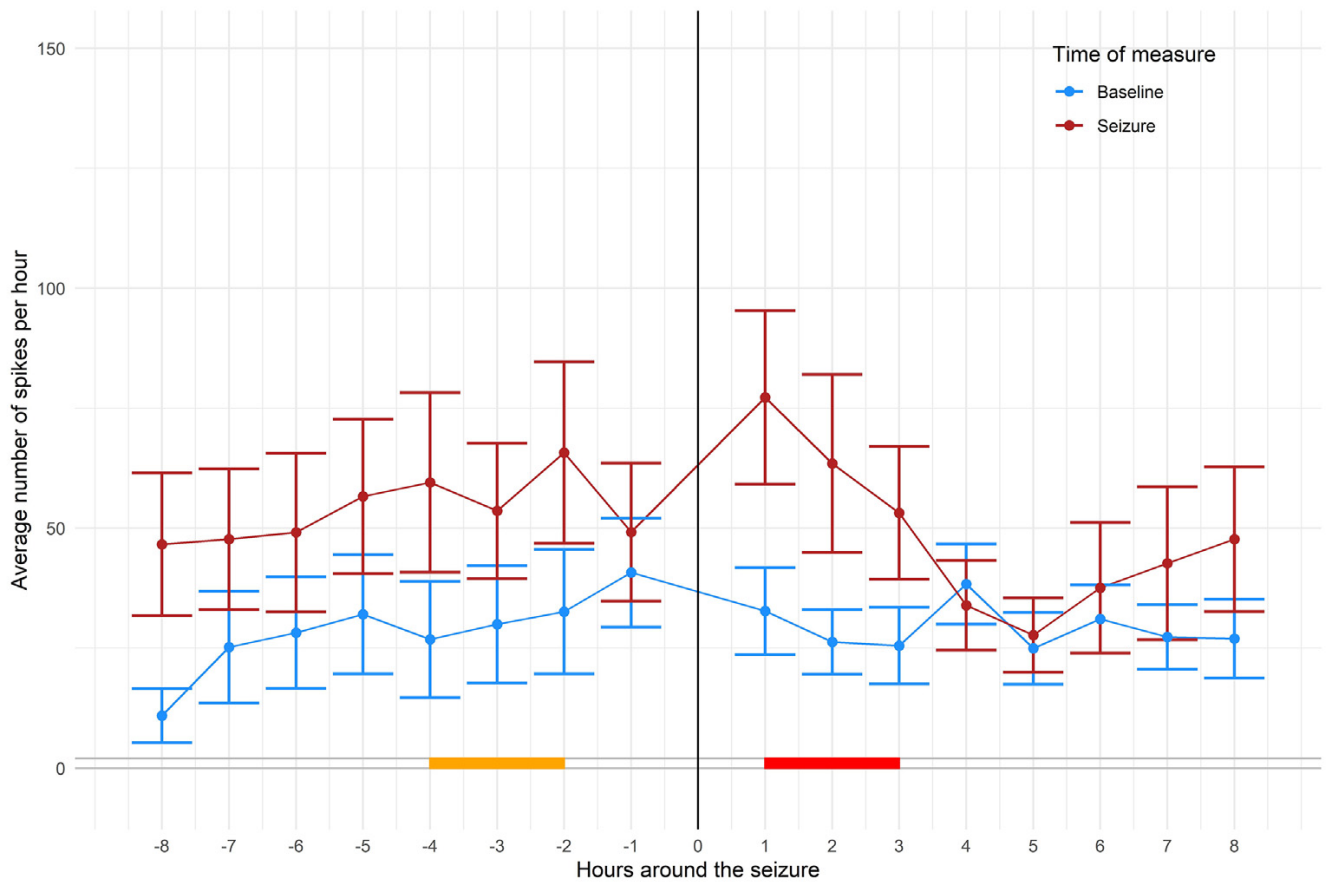
Evolution of spiking before and after seizure onset compared to baseline. A: Averaged spike activity ± standard error before and after the seizure (−8 h to +8 h) for each seizure of each patient (blue whiskers). Red whiskers: spike rate (SR) during baseline (same time of the day at day 1 on full medication). Yellow and red segments at the bottom indicates the periods of significant difference between baseline and peri-ictal periods. (For interpretation of the references to colour in this figure legend, the reader is referred to the web version of this article.)

Regarding the effect of ASM withdrawal, SR increased significantly during the day of lowest drug charge (Fig. 2) compared to baseline (t = 7.44; *p* < 0.001). This finding is in line with the analysis of the SR in the peri-ictal period. When comparing the latter period with the baseline, as shown in Fig. 3 the SR is constantly higher. A clustermass test highlighted a strong tendency to increase in the SR around seizure, in particular during 4 to 2 h before the seizure (F = 15.33; p = 0.05), extending up significantly to 3 h after the seizure (F = 21.32; p = 0.03), before decreasing and returning to baseline levels.

**Fig. 4:**
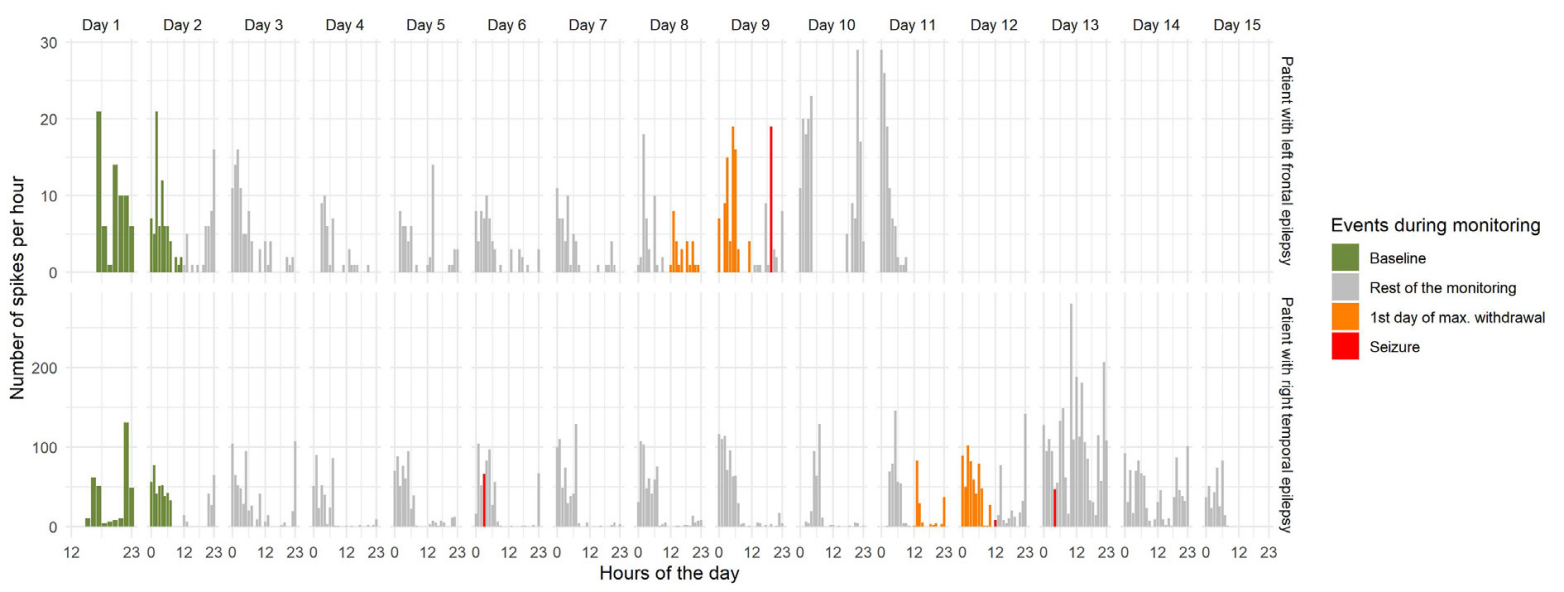
Examples of the evolution of spike rate during medication withdrawal in a left frontal lobe epilepsy patient and a right temporal lobe epilepsy patient.

Examples of SR profile of an extratemporal (frontal) lobe epilepsy patient and a temporal lobe epilepsy patient during medication withdrawal showing IED increase before and after the seizures are represented in Fig. 4.

## 4 Discussion

Our results suggest that interictal spikes increase significantly after ASM withdrawal, in particular in the hours around seizure occurrence. Using 38 scalp-electrodes, equally distributed over the scalp, and automatic spike counts of the entire video-EEG-monitoring, verified by expert EEG readers, helped to determine the exact spike activity per hour in relation to seizures and drug changes.

The significant increase in SR of the day of lowest medication load compared to day 1 (baseline) under full medication confirms the well-established notion that medication withdrawal is a reliable method to activate the epileptic focus in patients with pharmaco-resistant epilepsy. In patients with a first unprovoked seizure, recurrence risk is higher if the EEG contains epileptiform abnormalities (Berg and Shinnar, 1991, Bouma et al., 2016).

The relationship of IEDs and seizure risk in idiopathic generalized epilepsy (IGE) is relatively well established and here, EEG has shown its quality as excellent disease monitor (Wirrell, 2010). One of the reasons might be that the capture of bilateral discharges, the signature pattern in IGE, is less dependent on electrode count, i.e. IEDs can be noticed even with 8–16 electrodes.

IEDs have been described as an independent predictor for seizure recurrence after epilepsy surgery (Rathore and Radhakrishnan, 2010, Yu et al., 2010). Therefore, IEDs convey information on seizure risk in patients with chronic epilepsy. Moreover, there is considerable evidence that interictal spiking contributes to cognitive impairment, requiring also a close watch of IEDs and not only of seizure reports (Holmes, 2013).

Since the milestone study by Cook et al. (2013) it is well known that patient’s reports are highly unreliable.

Our results indicate that increased SR is associated with drug withdrawal in an individual patient, or, in clinical practice, with decreased medication intake, drug interference or efficiency, calling for drug level controls.

The SR increased 2 to 4 h consistently before seizure occurrence, if SR was compared with the same circadian period during baseline. There was a small dip in SR in the hour before the seizure occurred, which may explain the divergent findings of increase versus decrease of SR before seizures. However, this was not significant.

Most patients differed in their SR as a function of the time of the day. Our results suggest that increased spiking during susceptible periods of the day is a red flag for seizure occurrence, provided that baseline SR activity is known. A practical consequence of our findings could be that EEG controls of an individual patient should be performed always at the same time of the day to allow comparison.

There are several hypotheses on the role of interictal spikes. It has been proposed that spikes create a state of refractoriness in the epileptic network (Staley et al., 2001, Staley et al., 1998), transiently reducing the probability of other epileptiform discharges (de Curtis and Avanzini, 2001) and consequently also seizures. On the other hand, the synchronous synaptic activation and membrane depolarization that occur during spiking may also induce long-term increases of neuronal excitability in the epileptic focus (Bains et al., 1999), and consequently increases the probability of seizures (Staley et al., 2011). Changes in cortical excitability have also been shown with transcranial magnetic brain stimulation in patients with unilateral temporal lobe epilepsy before and after drug reduction. (Wright et al., 2006). Our results partially corroborate the results of Goncharova et al. 2016 (Goncharova et al., 2016), who found increased SR during periods of seizure occurrence. However, in their study medication withdrawal rather decreased the interictal spike activity. This may be explained by different analysis strategies: they excluded 6 h around seizure for analysis, whereas we excluded only the hour involving seizure. Moreover, the circadian aspect of spiking and seizure has not been taken into account.

Based on a milestone study on spike and seizure counts over several months and years through chronically implanted devices, it was shown that seizures and increased SR activity tend to occur during preferred phases in circadian and multidien rhythms (Baud et al., 2018). SR followed a 12-hours harmonic of the circadian rhythm and tended to be more frequent around seizure occurrence (Baud et al., 2018), corroborated by other observations (Cook et al., 2016, Karoly et al., 2017). These studies, together with our results, strongly suggest that increased spiking is a precursor phenomenon of upcoming seizures.

In this sense, interesting results showing how intrinsic excitability measures were negatively correlated with medication load, provide a step toward the development of a reliable quantitative measure of central effects of ASM in patients with epilepsy (Meisel et al., 2016), as well as HFO rates and mean duration may provide information to track drug load (Zijlmans et al., 2009).

Our study has several limitations. The low number of patients did not allow us to investigate correlations within the subgroups (ex. temporal versus extratemporal foci). The lack of standardized ASM withdrawal schema prohibited to determine the drug effect. Also, the effect of specific drugs on interictal SR could not be addressed: most patients were under polytherapy, and withdrawal was personalized (e.g. if lamotrigine controlled best the epilepsy, this drug was taken off first). Moreover, polytherapy included drugs of different half-lives, and we did not determine drug levels each day during monitoring. The effect of drug withdrawal varies with the type of ASM: withdrawal of sodium-channel blockers compared to drugs with other mode of actions are more likely followed by a major seizure increase or even bilateral tonic-clonic seizures. Such a study would need a very high number of patients, with currently 5–10 frequently used ASM and numerous combinations of drugs and permuations of the sequence of drug withdrawal (i.e., lamotrigine withdrawal before levetiracetam withdrawal or the other way around). However, most of our patients received treatment of drugs with different mode of actions, which probably levels out the effect of single drugs.

## 5 Conclusions

Our results corroborate the hypothesis that interictal spikes are a marker of cortical excitatory mechanisms increasing the likelihood of seizure. If spikes and seizures show similar probability distributions, both are not independent processes. IEDs could be a useful marker of seizure control also in patients with focal epilepsy, requiring regular routine EEGs and comparison with the patient’s baseline, obtained during the same time of EEG recording. Although the notion “we treat the patient and not the EEG” still prevails, neurologists and neuropediatricians should be alarmed if the EEG shows an increase of spikes. In contrast to IGE, adequate scalp coverage is much more crucial in focal epilepsy (Seeck et al., 2017). Longer EEGs with a higher likelihood of IEDs could allow objectifying IED activity through visual analysis or automatic spike counts, as in the present study. Future larger single- or multicentre trials are mandatory to determine the utility of such an approach and cut-offs of significant IED increases, which could be a first step towards personalized therapy for patients with epilepsy.

## 6 Data statement

Due to data protection reasons, data cannot be made publicly available. However, anonymized grouped data will be made available upon reasonable request to qualified investigators.

## 7 Role of the funding source

Author PDS, EM, and MS were supported by Swiss National Science Foundation (SNSF) CRS115-180365.

## 8 Disclosure

All authors have approved the final article.

## Declaration of Competing Interest

The authors declare the following financial interests/personal relationships which may be considered as potential competing interests: PDS and EM have any conflict of interest to disclose. MS, SV and PVM report that they are shareholders of Epilog NV (Ghent, Belgium).

## References

[b0005] Andersen N.B., Alving J., Beniczky S. (2010). Effect of medication withdrawal on the interictal epileptiform EEG discharges in presurgical evaluation. Seizure.

[b0010] Bains J.S., Longacher J.M., Staley K.J. (1999). Reciprocal interactions between CA3 network activity and strength of recurrent collateral synapses. Nat. Neurosci..

[b0015] Bates D., Mächler M., Bolker B., Walker S. (2015). Fitting linear mixed-effects models using lme4. J. Stat. Softw..

[b0020] Baud M.O., Kleen J.K., Mirro E.A., Andrechak J.C., King-Stephens D., Chang E.F., Rao V.R. (2018). Multi-day rhythms modulate seizure risk in epilepsy. Nat. Commun..

[b0025] Berg A.T., Shinnar S. (1991). The risk of seizure recurrence following a first unprovoked seizure: A quantitative review. Neurology.

[b0030] Bouma H.K., Labos C., Gore G.C., Wolfson C., Keezer M.R. (2016). The diagnostic accuracy of routine electroencephalography after a first unprovoked seizure. Eur. J. Neurol..

[b0035] Brooks M.E., Kristensen K., van Benthem K.J., Magnusson A., Berg C.W., Nielsen A., Skaug H.J., Mächler M., Bolker B.M. (2017). glmmTMB balances speed and flexibility among packages for zero-inflated generalized linear mixed modeling. R Journal..

[b0040] Cook M.J., O’Brien T.J., Berkovic S.F., Murphy M., Morokoff A., Fabinyi G., D’Souza W., Yerra R., Archer J., Litewka L., Hosking S., Lightfoot P., Ruedebusch V., Sheffield W.D., Snyder D., Leyde K., Himes D. (2013). Prediction of seizure likelihood with a long-term, implanted seizure advisory system in patients with drug-resistant epilepsy: a first-in-man study. Lancet Neurol..

[b0045] Cook M.J., Karoly P.J., Freestone D.R., Himes D., Leyde K., Berkovic S., O’Brien T., Grayden D.B., Boston R. (2016). Human focal seizures are characterized by populations of fixed duration and interval. Epilepsia.

[b0050] de Curtis M., Avanzini G. (2001). Interictal spikes in focal epileptogenesis. Prog. Neurobiol..

[b0055] Fox J., Weisbery S. (2019).

[b0060] Frossard J., Renaud O. (2021). Permutation tests for regression, anova, and comparison of signals: The permuco package. J. Stat. Softw..

[b0065] Goncharova I.I., Alkawadri R., Gaspard N., Duckrow R.B., Spencer D.D., Hirsch L.J., Spencer S.S., Zaveri H.P. (2016). The relationship between seizures, interictal spikes and antiepileptic drugs. Clin. Neurophysiol..

[b0070] Gotman J., Koffler D.J. (1989). Interictal spiking increases after seizures but does not after decrease in medication. Electroencephalogr. Clin. Neurophysiol..

[b0075] Gotman J., Marciani M.G. (1985). Electroencephalographic spiking activity, drug levels, and seizure occurrence in epileptic patients. Ann. Neurol..

[b0080] Holmes G.L. (2013). EEG abnormalities as a biomarker for cognitive comorbidities in pharmacoresistant epilepsy. Epilepsia.

[b0085] Karoly P.J., Nurse E.S., Freestone D.R., Ung H., Cook M.J., Boston R. (2017). Bursts of seizures in long-term recordings of human focal epilepsy. Epilepsia.

[b0090] Kuznetsova A., Brockhoff P.B., Christensen R.H.B. (2017). lmerTest package: tests in linear mixed effects models. J. Stat. Softw..

[b0095] Leguia M.G., Andrzejak R.G., Rummel C., Fan J.M., Mirro E.A., Tcheng T.K., Rao V.R., Baud M.O. (2021). Seizure cycles in focal epilepsy. JAMA Neurol..

[b0100] Ludwig B.I., Ajmone Marsan C. (1975). EEG changes after withdrawal of medication in epileptic patients. Electroencephalogr. Clin. Neurophysiol..

[b0105] Meisel C., Plenz D., Schulze-Bonhage A., Reichmann H. (2016). Quantifying antiepileptic drug effects using intrinsic excitability measures. Epilepsia.

[b0110] Ménétré, E., 2021. NPL: An incomplete toolkit for the researcher in NeuroPsycholinguistic, https://github.com/EricMenetre/NPL.

[b0115] Milligan N., Oxley J., Richens A. (1983). Acute effects of intravenous phenytoin on the frequency of inter-ictal spikes in man. Br. J. Clin. Pharmacol..

[b0120] Nash J.C., Varadhan R. (2011). Unifying optimization algorithms to aid software system users: optimx for R. J. Stat. Softw..

[b0125] R Core Team (2020).

[b0130] Rathore C., Radhakrishnan K. (2010). Prognostic significance of interictal epileptiform discharges after epilepsy surgery. J. Clin. Neurophysiol..

[b0135] Seeck M., Koessler L., Bast T., Leijten F., Michel C., Baumgartner C., He B., Beniczky S. (2017). The standardized EEG electrode array of the IFCN. Clin. Neurophysiol..

[b0140] Spencer S.S., Goncharova I.I., Duckrow R.B., Novotny E.J., Zaveri H.P. (2008). Interictal spikes on intracranial recording: Behavior, physiology, and implications. Epilepsia.

[b0145] Staley K.J., Longacher M., Bains J.S., Yee A. (1998). Presynaptic modulation of CA3 network activity. Nat. Neurosci..

[b0150] Staley K.J., Bains J.S., Yee A., Hellier J., Longacher J.M. (2001). Statistical model relating CA3 burst probability to recovery from burst-induced depression at recurrent collateral synapses. J. Neurophysiol..

[b0155] Staley K.J., White A., Dudek F.E. (2011). Interictal spikes: Harbingers or causes of epilepsy?. Neurosci. Lett..

[b0160] Wickham, H., Henry, L., 2019. tidyr: Easily Tidy Data with “spread” and “gather” Functions, https://cran.r-project.org/web/packages/tidyr/index.html.

[b0165] Wickham, H., François, R., Henry, L., Müller, K., 2019. A grammar of data manipulation, https://cran.r-project.org/web/packages/dplyr/index.html.

[b0170] Wickham, H., 2016. ggplot2 - Elegant Graphics for Data Analysis (2nd Edition). https://doi.org/10.18637/jss.v077.b02.

[b0175] Wilkus R.J., Green J.R. (1974). Electroencephalographic investigations during evaluation of the antiepileptic agent sulthiame. Epilepsia.

[b0180] Wirrell E.C. (2010). Prognostic significance of interictal epileptiform discharges in newly diagnosed seizure disorders. J. Clin. Neurophysiol..

[b0185] Wright M.-A.-S.-Y., Orth M., Patsalos P.N., Smith S.J.M., Richardson M.P. (2006). Cortical excitability predicts seizures in acutely drug-reduced temporal lobe epilepsy patients. Neurology.

[b0190] Yu H.-Y., Yen D.-J., Yiu C.-H., Lin Y.-Y., Kwan S.-Y., Chen C., Hsu S.P.C., Shih Y.-H. (2010). Postoperative interictal epileptiform discharge within 1month is associated with seizure recurrence after anterior temporal lobectomy. Epilepsy Behav..

[b0195] Zijlmans M., Jacobs J., Zelmann R., Dubeau F., Gotman J. (2009). High-frequency oscillations mirror disease activity in patients with epilepsy. Neurology.

